# Patterns of Genetic and Reproductive Traits Differentiation in Mainland vs. Corsican Populations of Bumblebees

**DOI:** 10.1371/journal.pone.0065642

**Published:** 2013-06-06

**Authors:** Thomas Lecocq, Nicolas J. Vereecken, Denis Michez, Simon Dellicour, Patrick Lhomme, Irena Valterová, Jean-Yves Rasplus, Pierre Rasmont

**Affiliations:** 1 Laboratoire de Zoologie, University of Mons, Mons, Belgium; 2 Evolutionary Biology and Ecology, Université Libre de Bruxelles, Brussels, Belgium; 3 Institute of Organic Chemistry and Biochemistry, Academy of Sciences of the Czech Republic, Prague, Czech Republic; 4 Institut national de la recherche agronomique, UMR Centre de Biologie pour la Gestion des Populations, Montferrier sur Lez, France; CNRS, France

## Abstract

Populations on islands often exhibit lower levels of genetic variation and ecomorphological divergence compared to their mainland relatives. While phenotypic differentiation in characters, such as size or shape among insular organisms, has been well studied, insular differentiation in quantitative reproductive traits involved in chemical communication has received very little attention to date. Here, we investigated the impact of insularity on two syntopic bumblebee species pairs: one including species that are phylogenetically related (*Bombus terrestris* and *B. lucorum*), and the other including species that interact ecologically (*B. terrestris* and its specific nest inquiline *B. vestalis*). For each bumblebee species, we characterized the patterns of variation and differentiation of insular (Corsican) vs. mainland (European) populations (i) with four genes (nuclear and mitochondrial, 3781 bp) and (ii) in the chemical composition of male marking secretions (MMS), a key trait for mate attraction in bumblebees, by gas chromatography-mass spectrometry (GC-MS). Our results provide evidence for genetic differentiation in Corsican bumblebees and show that, contrary to theoretical expectations, island populations of bumblebees exhibit levels of genetic variation similar to the mainland populations. Likewise, our comparative chemical analyses of MMS indicate that Corsican populations of bumblebees are significantly differentiated from the mainland yet they hold comparative levels of within-population MMS variability compared to the mainland. Therefore, insularity has led Corsican populations to diverge both genetically and chemically from their mainland relatives, presumably through genetic drift, but without a decrease of genetic diversity in island populations. We hypothesize that MMS divergence in Corsican bumblebees was driven by a persistent lack of gene flow with mainland populations and reinforced by the preference of Corsican females for sympatric (Corsican) MMS. The impoverished Corsican bumblebee fauna has not led to relaxation of stabilizing selection on MMS but to consistent differentiation chemical reproductive traits on the island.

## Introduction

Islands have always been of particular interest for evolutionary biologists and ecologists alike. Indeed, insularity offers unique opportunities to investigate a range of micro-evolutionary processes that precede macro-evolutionary events [1]. Insularity has often led to the same consequences in phylogenetically disparate groups of organisms, the so-called “island syndrome”. At species community level, islands are often characterized by depauperate fauna compared to the mainland [2]–[4], a phenomenon that might affect rates of inter-specific interactions, e.g. with closely-related taxa, or with natural enemies [5]. At the species level, island populations are often characterized by a series of genetic and phenotypic changes. Theory predicts that reduced immigration rates from mainland, founder events and genetic drift [2], [6] generally result in island populations being genetically impoverished compared to their mainland counterparts [7]–[9]. Furthermore, populations confined to geographically-isolated and contrasting habitats on islands often experience ecomorphological divergence from their mainland relatives. This divergence could result from genetic changes (founder effects or drift) and/or from post-colonization adaptive changes produced by different selection regimes compared to the adjacent mainland habitats [1], [2], [10]–[15].

The trend in insular populations to depart from mainland phenotypes is known to affect a wide range of characters (morphology, physiology, behavior) as well as life-history traits [3], [10], [16]–[22]. Consequently to phenotypic changes, a vast range of species that have evolved distinct island forms have sometimes been ascribed to their own taxonomic status (races, subspecies or sometimes even species) (e.g. [23]–[25]). Whether these phenotypic differences are genetically-based or reflect phenotypic plasticity (e.g. [26], [27]) has not been deeply investigated.

Phenotypic differentiation of insular populations in characters such as size (dwarfism vs. gigantism) or shape has been the focus of abundant research thus far (see [4], [15] and references therein). In contrast, insular differentiation in reproductive and quantitative traits such as sex pheromones has received comparatively far less attention (but see [28], [29]), despite their key role in the maintenance of reproductive isolation (e.g. [30]–[35]). Like most reproductive traits, courtship pheromones are shaped (i) by intraspecific interactions to maximize encounter rates among conspecific mates (sexual selection [36], [37]), and (ii) by interspecific interactions to maintain isolation barriers and decrease the likelihood of hybridization events among syntopic sister species [31], [37]–[39], and to minimize eavesdropping by potential predators or parasites [40], [41].

In this paper, we investigated the genetic and phenotypic consequences of insularity in Corsican bumblebees (*Bombus*, Hymenoptera, Apidae). We used a phylogenetic and phylogeographic approach based on sequence analyses of four genes (nuclear and mitochondrial) along with comparative chemical analyses of the differentiation patterns and natural variation of male marking secretions (MMS). The MMS are an important reproductive trait in male bumblebees that determines the attraction of conspecific virgin females. We focused on two syntopic species pairs, one including two sister species (*Bombus (Bombus) terrestris* (L.) and *B. (Bombus) lucorum* (L.)) and the other with two ecologically interacting species (*B. terrestris* and its specific nest inquiline *B.* (*Psithyrus*) *vestalis* (Fourcroy)). Specifically, we asked the following questions: (i) are Corsican bumblebees genetically and chemically differentiated from their conspecific European mainland populations? (ii) do Corsican bumblebees exhibit particular changes in the overall genetic diversity and male marking secretions variability compared to their conspecific European mainland populations (i.e., the relaxed selection hypothesis)?

## Materials and Methods

### Studied insular system and species

Corsica (8680 km^2^) is a continental, mountainous Mediterranean island located 160 km from the French coast, 12 km from Sardinia, 82 km from the Italy, and nearly 50 km from the Island of Elba, which is located 10 km off the coast of Italy. The island is a well-known biodiversity hotspot [42]–[45] that hosts a high diversity of endemic species [43], [45], [46] and also represents a major glacial refuge in Europe (e.g. [47]).

**Figure 1:**
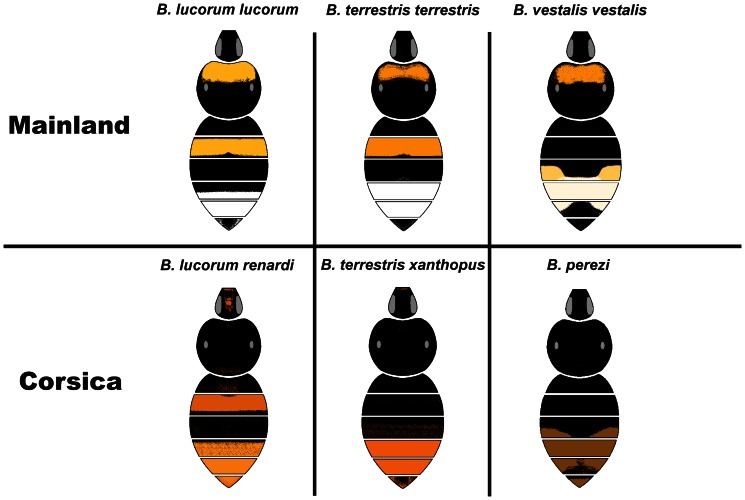
Morphological differentiation between mainland and Corsican taxa of the three bumblebee species investigated. The female color patterns are represented. *Bombus lucorum* and *B. terrestris* are sister species in the subgenus *Bombus* and *B. vestalis* is a specific nest inquiline of *B. terrestris*.

Corsican endemics differ in their biogeographic origin and in the way they colonized Corsica. Colonization by bumblebees may have occurred during the last Ice Age (Würm, 115 000–10 000 years) from Italy via the present Tuscan Archipelago [48], [49]. Six endemic taxa are recorded from Corsica [48]. *Bombus lucorum* is a species widely distributed in the Palaearctic [50] while *B. terrestris* and *B. vestalis* are restricted to the West-Palaearctic region [51], [52]. The nearest Corsican taxa of these species are: (i) *B. lucorum renardi* Radoszkowski 1884, (ii) *B. terrestris xanthopus* Kriechbaumer 1870, and (iii) *B. perezi* (Schulthess-Rechberg 1886), respectively. The taxonomic status of the Corsican populations is still not clearly defined [48], [51], [53]–[56]. Therefore, comparisons have been made between the Corsican taxa and their continental populations (for *B. terrestris* and *B. lucorum*) or their most closely-related mainland species (for *B. vestalis* and *B. perezi*) (Table 1). Corsican populations of these species are morphologically distinct [48] with specific color patterns, black hairs and a red-brownish tail (Fig. 1). Previous studies on *B. terrestris* have shown that Corsican populations are genetically differentiated from those on the mainland [57] and exhibit a distinct diapause duration [58]. Likewise, Corsican populations of *B. perezi* are unique in being adapted to the winter phenology of their host, *B. terrestris* [48], [59], contrary to continental populations of *B. vestalis*.

**Table 1:** Corsican taxa and their continental populations (for *B. terrestris* and *B. lucorum*) or their most closely-related mainland species (for *B. vestalis* and *B. perezi*).

Corsican taxa	Continental Counterparts
*B. lucorum renard*i Radoszkowski 1884	*B. lucorum lucorum* (L.)
*B. terrestris xanthopus* Kriechbaumer 1870	*B. terrestris dalmatinus* Dalla Torre 1882 (Eastern Europe)
	*B. terrestris lusitanicus* Krüger 1956 (Iberian Peninsula)
	*B. terrestris terrestris* (L.) (Western Europe)
*B. perezi* (Schulthess-Rechberg 1886)	*B. vestalis* (Fourcroy 1785)

Courtship signals of male bumblebees include both behavioral and chemical features (see [60] for a review). Most bumblebee males patrol along paths where they scent-mark objects with species-specific secretions (male marking secretion, MMS) that attract conspecific virgin females [61], [62]. The height and localization of marked objects differ among species [63]. Female bumblebees also produce sex pheromones, which elicit male mating behavior and which are involved in species recognition [64]. The MMS, the path configuration and possibly also female sex pheromones are involved in pre-mating recognition. Here, we focus on the most studied trait, the MMS, which consists of a complex mixture of (mainly aliphatic) compounds, with several major components (e.g. ethyl tetradecenoate in *B. lucorum* [65]) and with intraspecific variation (e.g. [56], [66], [67]). MMS are synthesized *de novo* by cephalic labial glands [68], [69] and the chemical composition is not affected by environmental conditions, diet or imprinting [68].

### Sampling

**Figure 2:**
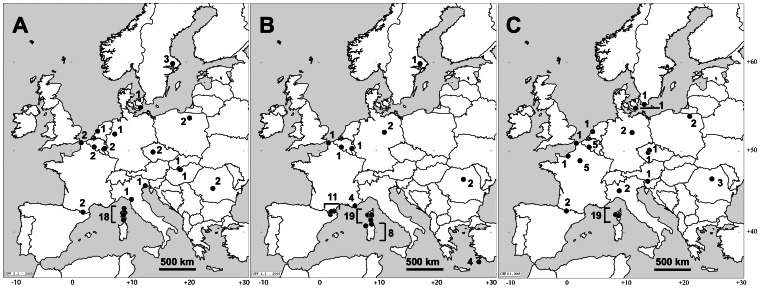
Sample maps of the three bumblebee models. A = *B. lucorum*; B = *B. terrestris*; C = *B. vestalis*. The number near sample sites is the number of samples collected at each sample sites.

We sampled 57 males of *B. terrestris* (L.), 42 males of *B. lucorum* (L.), and 48 males of *B.* (*Psithyrus*) *vestalis-perezi* (Fourcroy) in Corsica, as well as in localities spread across European mainland and Sardinia (Fig. 2, Table S1). Males were killed by exposing them to freezing conditions, at −20°C. The MMS were extracted in 200 µl *n*-hexane from dissected cephalic labial glands or entire cut heads following De Meulemeester et al. [70]. Vials containing the solvent and sample were kept for 24 h at room temperature (20°C) to fulfill the extraction [70]. Beheaded bodies were preserved in 99% ethanol for DNA extraction. All samples were stored at −40°C prior to the analyses. Studied samples did not involve endangered or protected species. Permissions for collection of Corsican samples from were obtained via the *Office de l'Environnement de la Corse* and the *Direction Régionale de l'Environnement de Corse*. Collecting samples from lands of the *Office National des Forêts*, required permission, and this was obtained from the *Office National des Forêts Méditerranée*. No specific permits were required for the other described field studies as collection did not occur in privately-owned or protected locations.

### Molecular analyses

#### Gene selection, DNA preparation, amplification and sequencing

We sequenced four genes commonly used to study interspecific and intraspecific relationships among bumblebees (e.g. [57], [71]–[73]): mitochondrial cytochrome oxidase 1 (COI), cytochrome b (Cytb), nuclear protein-coding gene elongation factor-1 alpha, F2 copy (EF-1α) and phosphoenolpyruvate carboxykinase (PEPCK). The usefulness of nuclear gene sequences in discriminating population-level divergence appears limited (even intron regions) by the substantially greater coalescence time (and associated variance) compared to mitochondrial genes. However, according to the short life cycle (one or two generations every year) of bumblebees, we tested putatively variable nuclear genes.

Total DNA extraction was performed using a QIAGEN DNeasy® Tissue Kit (Quiagen Inc., Valencia, CA). Legs were removed from the specimen, crushed using liquid nitrogen, and digested for four hours in proteinase K at 56°C. Voucher specimens and PCR products used in molecular investigation were deposited at the University of Mons (Belgium).

Polymerase chain reaction (PCR) amplifications were carried out for all genes and all samples using primer pair Jerry/Pat [74] for COI, CB1/CB2 [75] for Cytb, F2-ForH/F2-RevH2 [76] for EF-1α, and FHv4/RHv4 [72]for PEPCK. PCR amplifications were carried out by initial denaturing for three minutes at 94°C, 35 (COI, EF-1α) or 40 (Cytb and PEPCK) cycles of one minute denaturing at 94°C, one minute annealing at 51°C (COI), 50°C (Cytb), 54°C (EF-1α) or 48.5°C (PEPCK), two minutes elongation at 72°C and a final extension for ten minutes at 72°C). Genes were sequenced with an ABI 3730XL sequencer (Applied Biosystems, Foster City, CA, USA) or by GENOSCOPE (*Centre National de Séquençage*; Evry, France). Both strands of each PCR product were sequenced.

Consensus sequences were computed with CodonCode Aligner 3.0.1. As bumblebee males are haploid [77], there is no uncertainty in the consensus sequences. The bumblebee origin of each sequence was checked with BLAST 2.2.20 [78]. The alignment was performed by MAFFT ver.6. (using FFT-NS-2 algorithms, default parameters) [79]. The data matrix was computed on Mesquite 2.74 (build 486) [80]. Translation to proteins (using the *Drosophila* mitochondrial DNA genetic code or Universal genetic code) was performed with Mesquite. Sequences are available on Genbank, accessions JQ820504 to JQ821313 (Table S1).

The final molecular dataset spanned 3781 aligned nucleotides: 849 bp from COI (218 parsimony informative sites (PIS)), 459 bp from Cytb (112 PIS), 773 bp from EF-1α F2 copy containing a ∼200 bp intron (92 PIS), and 859 bp from PEPCK (117 PIS). Haplotype numbers are 1) *B. lucorum*: three for COI, two for Cytb, one for EF-1α, and four for PEPCK; 2) *B. terrestris*: 14 for COI, seven for Cytb, one for EF-1α, and seven for PEPCK; 3) *B. vestalis*: six for COI, four for Cytb, three for EF-1α, and three for PEPCK.

#### Phylogenetic and population structure analyses

We performed phylogenetic and phylogeographic analyses to investigate the extent of genetic differentiation/isolation of Corsican populations of each bumblebee species. In phylogenetic analyses, we analyzed each gene independently and combined (all mitochondrial genes and all nuclear genes) using maximum parsimony (MP), maximum likelihood (ML) and Bayesian methods (MB). A test of saturation was applied to each fragment in PAUP* 4.0b 10 [81]. The Incongruence Length Difference (ILD) test [82], as implemented by PAUP*, was used to evaluate incongruence between genes. Trees were rooted with basal subgenus of the bumblebee clade: *Mendacibombus* (*Bombus mendax* Gerstaecker 1869 and *Bombus shaposnikovi* (Skorikov 1910)) [72], [83], [84]. Other closely related taxa were included in our analyses: *Bombus (Bombus) affinis* Cresson 1863, *Bombus (Psithyrus) bohemicus* Seidl 1937, *Bombus (Bombus) hypocrita* Pérez 1905, *Bombus (Bombus) ignitus* Smith 1869, and *Bombus (Bombus) sporadicus* Nylander 1848.

Heuristic searches were performed in MP using 1000 random additions and tree bisection reconnection branch swapping, keeping the best trees only. Gaps were regarded as a fifth state. Majority rule 50% consensus (MJ50) trees were constructed from analyses of individual genes and from all genes combined using parsimony criteria in PAUP* 4.0b 10 for equally-weighted MP analyses. Clade support values [85] were estimated using nonparametric bootstrapping in PAUP* (10000 replicates, 1000 random additions, 500 trees saved per replicate).

ML analyses were conducted in GARLI 2.0 [86]. Each gene was partitioned to explore the best substitution model: 1) EF-1α into two exons and one intron; 2) PEPCK into two exons and two introns; 3) COI, Cytb, each EF-1α exon, and PEPCK exons by base position (1^st^, 2^nd^ and 3^rd^). The best fitting substitution models were chosen with jModeltest [87] using the Akaike information criteria corrected for small sample sizes (AICc) [88] for each dataset. The chosen models were: 1) For COI: TIM2+I (1^st^) and TPM2uf+I+G (2^nd^ and 3^rd^); 2) For CytB: TPM1uf+G (1^st^), TrN+I+G (2^nd^) and HKY+G (3^rd^); 3) For EF-1α exon 1: F81 (1^st^ and 2^nd^) and TIM2+G (3^rd^); 4) For EF-1α intron: TIM1+G; 5) For EF-1α exon 2: TrN (1^st^), JC (2^nd^) and TPM2uf (3^rd^); 6) For PEPCK intron 1: TPM2uf+G; 7) For PEPCK exon 1 HKY (1^st^), K80 (2^nd^) and TPM3uf (3^rd^); 8) For PEPCK intro 2 TIM3+G; 9) For PEPCK exon 2: K80 (1^st^ and 2^nd^) and TIM3+G (3^rd^). A random starting tree and the automated stopping criterion (stop when the ln score remained constant for 20000 consecutive generations) were used. Ten independent runs in GARLI were carried out for each gene and for the combined data; the topology and −ln L were identical among replicates. The highest likelihood of one of those runs was retained. Statistical confidence in nodes was evaluated using 1000 non-parametric bootstrap replicates [85] using the automated stopping criteria set at 10000 generations. More bootstrap replicates could not be performed because it would have required unpractical computing times. Topologies with bootstrap values ≥70% were considered well supported [89].

Bayesian analyses (MB) were carried out using MrBayes 3.1.2 [90]. The model selection process was the same as that for ML analysis. Selected models which are not implemented in MrBayes were substituted by the closest over-parameterized model [91]. The TIM1, TIM2, TIM3, TPM1uf, TPM2uf, TPM3uf and TrN substitution models were replaced by the GTR model. The proportion of invariable sites (I) and gamma distributed rates (G) defined in jModeltest were conserved in all models. Moreover, genes were analyzed individually and collectively. Five independent analyses were carried out for each gene and for the combined data (10 million generations, four chains with mixed-models, default priors, saving trees every 100 generations). The analyses were stopped after checking convergence between runs using the average standard deviation of split frequencies and by plotting likelihood values across generations using Tracer 1.4 [92]. The first one million generations were discarded as burn-in. The phylogeny and posterior probabilities were then estimated from the remaining trees and a majority-rule 50% consensus tree was constructed. Topologies with posterior probabilities ≥0.95 were considered well supported [93].

Haplotype networks were constructed using the median joining method in Network 4.6.1.0 (available at http://www.fluxus-engineering.com) for each gene and for each species. The median-joining method uses a maximum parsimony approach to search for all the shortest phylogenetic trees for a given data set [94]. To reconstruct the network, we weighted transversions twice as high as transitions.

We assessed patterns of genetic structure (a) only in mainland populations (b) in all sampled populations to evaluate whether or not the Corsican populations were genetically isolated from the mainland. We used the Analysis of Molecular Variance (AMOVA) [95] in Arlequin ver. 3.5 [96] with 100,000 random permutations. The estimation of F_st_ statistics considering one group of populations was used to assess potential genetic structure on the mainland and to determine if the addition of Corsican populations in the mainland group increases the population genetic structure of the species [97].

#### Genetic diversity within populations

Genetic diversity was estimated using nucleotide diversity (π) [98] and haplotype diversity (*h*) [99] using DnaSP [100] for each gene and for each species.

### Male marking secretion analyses

#### Chemical analyses

The composition of MMS was determined by gas chromatography-mass spectrometry (GC-MS) on a Finigan GCQ with a DB-5 ms non-polar capillary column (5% phenyl (methyl) polysiloxane stationary phase; 30 m×0.25 mm×0.25 µm) and an ion trap in electron impact mode “full scan (300–600)”. We used a splitless injection mode (220°C) and helium as carrier gas (50 cm/s). The temperature program of the column was set to 70°C for two minutes and then increased at a rate of 10°C by minute to 320°C. The temperature was then held at 320°C for five minutes. Compounds were identified in Xcalibur™ using their mass spectra compared to those at National Institute of Standards and Technology library (NIST, U.S.A) using NIST MS Search 2.0. The double bond positions were determined i) from mass spectra of dimethyl disulphide adducts of unsaturated components (reaction time: 4 h) and ii) by chemical ionization with acetonitrile as a reaction gas. The products were analyzed by GC-MS using the same temperature program as for original extracts. An ion trap GC-MS instrument (Varian Saturn, 2000) was used for chemical ionization.

All samples were analyzed using a gas chromatograph Shimadzu GC-2010 with a SLB-5 ms non-polar capillary column (5% diphenyl/95% dimethyl siloxane; 30 m×0.25 mm×0.25 µm) and a flame ionization detector. The chromatographic conditions were the same as those abovementioned. The peak areas of compounds were quantified using GCsolution Postrun (Shimadzu Corporation) with automatic peak detection and noise measurement. Relative amounts (RA in %) of compounds in each sample were calculated by dividing the peak areas of compounds by the total area of compounds in each sample using GCsolution Postrun (Shimadzu Corporation) with automatic peak detection and noise measurement. We did not use any correction factor to calculate the RA of individual compounds. All compounds for which RA were recorded as less than 0.1% for all specimens were discarded [101]. The data matrix for each species was elaborated with the relative proportion of each compound for each individual. Data matrices have been deposited in Tables S2, S3, S4.

#### Comparative Statistical Analyses Corsica versus mainland

All statistical analyses were performed using R [102] to detect MMS differentiations between insular and continental populations. Three groups were defined; each group included one Corsican endemic and its continental counterpart(s). Data consisting of the relative proportion of all compounds were transformed (log (x-1)) to reduce the great difference of abundance between highly and slightly concentrated compounds, and we then standardized (mean = 0, standard deviation = 1) the data matrix to reduce the sample concentration effect.

Clustering methods have already proved useful to detect and test differentiation among *Bombus* MMS (e.g. [73], [103]), so we used this approach to detect distance among taxa. Three additional matrices were computed: Euclidean, Pearson Phi Correlation, and Manhattan. Three clustering methods were used for each association matrix: single, complete, ward and UPGMA (R-package ape, [104]). We assessed the uncertainty in hierarchical cluster analysis using *p*-values calculated via multiscale bootstrap resampling with 50,000 bootstrap replications (R-package pvclust, [105]). When we detected MMS differentiations between Corsican and continental populations in clustering analyses, we assessed results by performing multiple response permutation procedure (MRPP) and a perMANOVA, i.e. a permutation-based version of the multivariate analysis of variance (MANOVA) (R-package vegan, [106]). The MRPP is a nonparametric, multivariate procedure that tests the null hypothesis of no difference between groups. MRPP has the advantage of not requiring distributional assumptions (such as multivariate normality and homogeneity of variances). A perMANOVA analysis was performed using the Bray-Curtis similarity matrix and 1000 permutations. Like conventional analyses of variances, the perMANOVA calculates an F statistic by taking the ratio of among group sums of squares to within group sums of squares. The perMANOVA is robust to violations of homogeneity of dispersion when the groups' sample sizes are equal. The returned *p*-value is used to detect significant differences which reflect changes in abundance and chemical composition of the MMS.

#### Chemical variability of MMS in Corsica vs. mainland populations

To evaluate differences in the compositional variability of MMS between Corsica and the mainland, we used a distance-based test for multivariate homogeneity of group dispersions (MHGH) for a one-way ANOVA design [107]. The procedure first calculates the Euclidean distances between MMS composition and respective group centroids (Corsica vs. mainland) on Pearson Phi Correlation matrices (R- package vegan [106]). To test if one group is more variable than the other, the magnitudes of these distances are then compared between groups using ordinary ANOVA. Moreover, a permutation test was run to generate a permutation distribution of F under the null hypothesis of no difference in dispersion between groups (R- package vegan [106]).

#### Interspecific chemical distance

To examine changes in intra- and inter-specific MMS variability between mainland and Corsican populations (i.e. the relaxed selection hypothesis), we compared the inter-specific chemical distance in Corsica vs. the mainland in R. We computed the Pearson Phi Correlation distance matrix for each pair of species (*B. lucorum* vs *B. terrestris*, *B. lucorum* vs *B. vestalis-perezi* and *B. terrestris* vs *B. vestalis-perezi*) among Corsican individuals and among mainland individuals. We then computed the mean distance of each individual to all heterospecific individuals on Corsica and on the mainland. Based on these mean distances, we performed Student's *t*-test with the null-hypothesis of no-difference in chemical interspecific distances between Corsica and mainland. When the *t*-test assumptions (independent observations, residues normality (Shapiro-Wilk test) and homoscedasticity (Bartlett's test)) were not met (p-values<0.05 for Shapiro and Bartlett's tests), equivalent non-parametric tests (Wilcoxon signed-rank test) were conducted.

## Results

### Genetic and chemical differentiation of the insular populations

**Figure 3:**
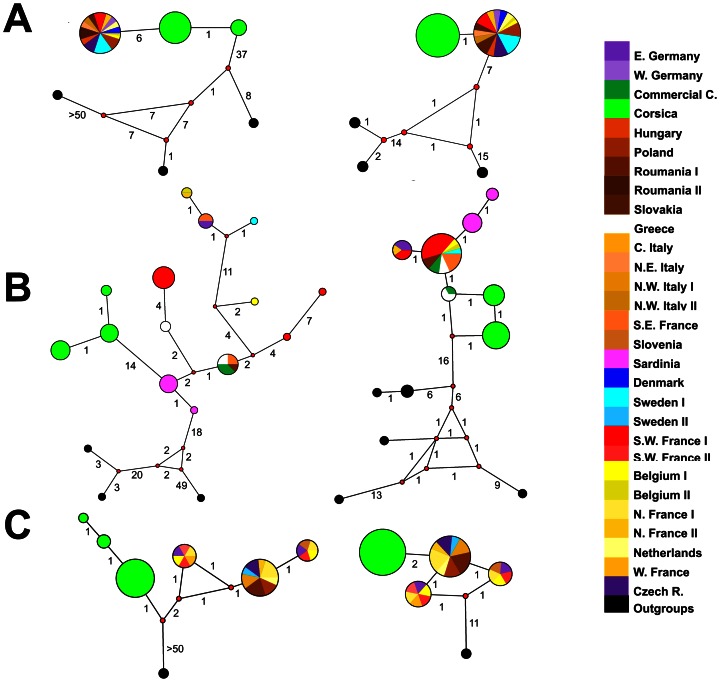
Median-joining network of haplotypes found in 4 model species ranges based on COI (on the left) and Cytb (on the right). A = *B. lucorum*; B = *B. terrestris*; C = *B. vestalis*. The sizes of the circles are proportional to frequencies of haplotypes. The red points on the lines represent undetected/extinct intermediate haplotype states. The numbers on the lines represent the number of mutations between two close haplotypes. The black circles are outgroups (see Table S1).

All phylogenetic analyses (maximum parsimony (MP), maximum likelihood (ML) and Bayesian methods (MB)) performed on the same dataset led to similar tree topologies and to identical relationships between Corsican and continental taxa. Mitochondrial (COI, Cytb and COI+Cytb) and nuclear (EF-1α, PEPCK and EF-1α+PEPCK) datasets produced different topologies. Mitochondrial datasets showed similar and well resolved topologies. Phylogenetic analyses based on nuclear datasets recovered all deep hierarchical levels relationships among subgenera and between species but EF-1α failed to discriminate between *B. lucorum* and *B. terrestris* (see supplementary trees available at TreeBase (study accession number S14022). Identical results were obtained using Network analyses (Fig. 3).

Analyses of genetic differentiation between populations of Corsican and mainland bumblebee taxa indicated a mitochondrial divergence but no nuclear differentiation. With the exception of *B. lucorum*, phylogenetic analyses of mtDNA failed to resolve continental and Corsican populations in two well-supported monophyletic groups. Mitochondrial haplotypes networks clearly illustrate that Corsican and continental populations form discrete groups. These findings are confirmed by strong mitochondrial genetic structure detected by AMOVA tests (F_st_>0.72, *P* -value<0.01; Table 2).

**Table 2:** Results of the AMOVA analysis using mainland populations alone and with insular populations.

		F_ST_			
Species	Origin	COI	EF1A	Cytb	PEPCK
***B. lucorum***	**Continent**	0	0	0	ns
	**Continent+Corsica**	0.93825	0	1	ns
***B. terrestris***	**Continent**	0.63681	0	ns	0.33551
	**Continent+Corsica**	0.83433	0	0.78066	ns
	**Continent+Sardinia**	0.68882	0	0.60738	0.40192
***B. vestalis***	**Continent**	ns	ns	ns	ns
	**Continent+Corsica**	0.81531	0.60479	0,78464	0.47727

**Only Fst value of the AMOVA tests with p-values<0.01 are represented (ns = non significant).**

We detected 55 chemical compounds in the MMS of *B. lucorum*, 105 in *B. terrestris* and 56 in *B. vestalis-perezi* (Tables S5, S6, S7). The main compounds (median>10%) of *B. lucorum* were aliphatic esters (ethyl tetradecenoate and hexadecyl tetradecenoate). *B. vestalis* secreted isoprenoids (geranylcitronellol and geranylcitronellyl acetate) and aliphatic aldehydes (icosadienal) as main compounds (median>10%). Corsican *B. lucorum* and *B. perezi* have the same main compound as their continental counterparts but different compounds present in smaller relative amounts (Tables S2 and S4). The MMS composition of continental *B. lucorum* and *B. vestalis* is very similar to previous studies [65], [108]. The main compounds of *B. terrestris* were (i) 2,3-dihydrofarnesol in *B. terrestris dalmatinus* and *B. terrestris sassaricus*, (ii) dihydrofarnesyl dodecanoate in *B. terrestris terrestris* and *B. terrestris lusitanicus*, (iii) tricosane for Corsican *B. terrestris xanthopus*. Corsican *B. terrestris* differed also in small compounds (Table S3). The MMS composition for mainland and Sardinian *B. terrestris* were similar to those reported by Coppée [56].

**Figure 4:**
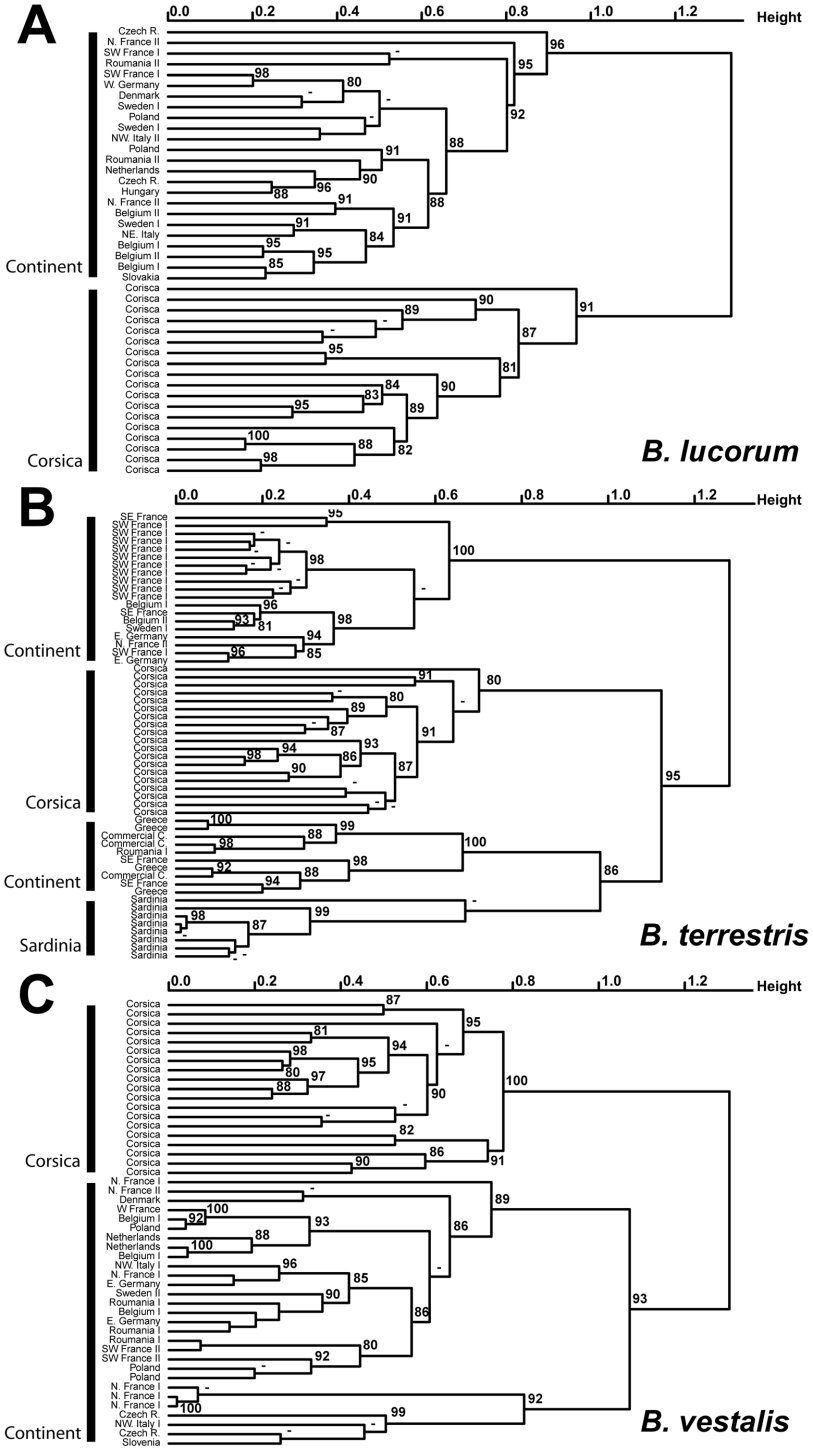
Unweighted pair group method with arithmetic mean (UPGMA) cluster based on a correlation matrix calculated from the matrix of male marking secretion. A = *B. lucorum* (55 compounds X 42specimens); B = *B. terrestris* (105 compounds X 56 specimens); C = *B. vestalis* (56 compounds X 48 specimens). Values above branch represent multiscale bootstrap resampling (only values >80% are given).

The statistical analyses of the MMS (MMS composition see Tables S2, S3, S4, S5) indicate that Corsican individuals differed from their conspecific mainland populations in all clusters, irrespective of the distance matrices and clustering methods (Fig. 4; only the UPGMA cluster based on the Phi Correlation matrix is shown). This chemical differentiation of Corsican taxa and samples is supported by high values of multiscale bootstrap resampling (>80%) (Fig. 4). PerMANOVA tests confirmed differentiation between Corsican and mainland populations for all species: *B. lucorum* (MRPP: T = 0.2869, A = 0.2405, *P*-value<0.01; perMANOVA: DF = 1, F = 30.95, *P*-value<0.01), *B. terrestris* (MRPP: T = 0.3023, A = 0.51, *P*-value<0.01; perMANOVA: DF = 1, F = 26.141, *P*-value<0.01) and *B. vestalis-perezi* (MRPP: T = 0.3782, A = 0.1543, *P*-value<0.01; perMANOVA: DF = 1, F = 17.705, *P*-value<0.01).

### Genetic diversity and chemical variability in insular populations

Nucleotide and haplotype diversities (π and *h*, respectively) recorded in Corsican and continental samples were globally low (Tables 3 and 4). The values of these parameters differed between Corsican and mainland populations (Tables 3 and 4) but the nucleotide and haplotype diversities were not always lower in Corsica compared to the mainland. We obtained higher or identical π and *h* values in Corsica compared to the mainland for all genes in *B. lucorum*, and 2) lower or similar π and *h* values in Corsica compared to the mainland for all genes for *B. terrestris* and *B. vestalis*.

**Table 3:** Haplotype diversity observed among individuals of three species groups present on island or on mainland.

		h			
Species	Origin	COI	EF1A	Cytb	PEPCK
**B. lucorum**	**Continent**	0 (0)	0 (0)	0 (0)	0.159 (0.094)
	**Corsica**	0.366 (0.1264)	0 (0)	0 (0)	0.778 (0.045)
**B. terrestris**	**Continent**	0.816 (0.045)	0 (0)	0.439 (0.097)	0.637 (0.080)
	**Corsica**	0.620 (0.061)	0 (0)	0.491 (0.068)	0.608 (0.089)
	**Sardinia**	0.250 (0.180)	0 (0)	0.429 (0.169)	0 (0)
**B. vestalis**	**Continent**	0.591 (0.072)	0.340 (0.090)	0.616 (0.064)	0.502 (0.040)
	**Corsica**	0.292 (0.127)	0.343 (0.128)	0 (0)	0.515 (0.052)

***h*** **is the haplotype diversity and the values in brackets are the standard deviations.**

**Table 4:** Nucleotide diversity observed among individuals of three species groups present on island or on mainland.

		π			
Species	Origin	COI	EF1A	Cytb	PEPCK
***B. lucorum***	**Continent**	0	0	0	0.00037 (0.00022)
	**Corsica**	0.00043 (0.00015)	0 (0)	0 (0)	0.00145 (0.00017)
***B. terrestris***	**Continent**	0.01286 (0.00154)	0 (0)	0.00103 (0.00026)	0.00112 (0.00023)
	**Corsica**	0.00085 (0.00014)	0 (0)	0.00107 (0.00015)	0.00165 (0.00038)
	**Sardinia**	0.00029 (0.00021)	0 (0)	0.00093 (0.00037)	0 (0)
***B. vestalis***	**Continent**	0.00120 (0.00021)	0.00044 (0.00012)	0.00157 (0.00023)	0.00058 (0.00005)
	**Corsica**	0.00045 (0.00022)	0.00045 (0.00014)	0 (0)	0.00059 (0.00006)

**π is the nucleotide diversity and the values in brackets are the standard deviations.**

The comparison of the MMS variability between Corsican and continental populations showed that there was a significant difference for *B. terrestris* (larger for mainland populations: AMOVA of multivariate dispersion *P* -value<0.01, permutation test of multivariate dispersion *P* -value<0.01) but not for other species: *B. lucorum* (AMOVA *P* -value = 0.249, permutation test p-value = 0.253) and *B. vestalis* (AMOVA *P* -value = 0.213, permutation test *P* -value = 0.213)

### Chemical interspecific distances in insular and continental communities

Interspecific distance between each species pair among Corsican individuals and among mainland individuals showed a larger interspecific distance in continental individuals (Distance *B. lucorum* – *B. terrestris*: t = −3.8395, df = 88, *P* -value<0.01; Distance *B. terrestris* – *B. vestalis*: W = 227, *P*-value<0.01; Distance *B. lucorum* – *B. vestalis*: W = 0, *P* -value<0.01).

## Discussion

### Genetic divergences

Most island populations experience reduced gene flow with the mainland [2]. The reduced gene flow can lead to genetic differentiation [3] and changes in genetic diversity [7]. The island populations diverge over time due to persistent genetic drift and/or changes in selection pressures compared to the mainland. Genetic differentiation of insular populations is common in birds (e.g. [109]), frogs (e.g. [110]), lizards (e.g. [111]), flying mammals (e.g. [112]), land insects (e.g. [113]), flying insects (e.g. [114]), and other bumblebee species (e.g. [57], [115], [116]). Island populations generally exhibit a lower genetic diversity compared to mainland populations because of genetic drift, founder effects and inbreeding, especially in small subpopulations [117]. The decrease of genetic diversity has also been reported in different groups of organisms [7], [118], [119]. Frankham [7] showed that a significant majority of island populations presented less genetic variability than their mainland counterparts, with an average reduction of 29%. This author noticed that the reduction in genetic variation was similar in island endemic and in non-endemic island populations in insects.

Here we show that geographic isolation has driven Corsican bumblebees to a significant degree of genetic differentiation (see also [57]). Genetic divergences between Corsican and continental populations are only observed in mitochondrial genes. The larger potential flight distance of males (which do not transmit their mitochondrial genome) compared to females cannot explain this result because the distance between Corsica and the mainland is larger than the potential flight distance of both sexes [120], [121]. This difference is presumably the consequence of the substantially greater coalescence time (and associated variance) of nuclear genes as compared to mitochondrial genes [122].

Contrary to predictions outlined by the island syndrome [119], there is no general trend of drastic loss of genetic diversity in Corsica compared to the mainland. High migration rates and separate migrations from differentiated mainland populations are generally invoked to explain why some island populations have similar genetic variation to mainland populations [7]. However, we hypothesize that the results presented here could be explained by particular events on the mainland rather than on the island. Indeed, the unexpected low genetic diversities (low π and low or intermediate *h*) on the mainland (comparison with other insects, e.g [123], [124]) despite the extensive sampling across species distribution could reflect a strong and prolonged bottleneck, or a recent population expansion from a small number of founder individuals [125]. This is most likely driven by past population bottlenecks during Quaternary glacial events as observed in several European species (e.g. [125]–[127]). The relatively high level of genetic diversity in Corsica might therefore suggest that the island has acted as a hotspot of intraspecific biodiversity, e.g. as a refuge during the glaciations as observed in other islands such as Sicily for rodents [128] and parasitic nematodes [129] or in Macaronesia for bryophytes [130]. Further studies of these patterns of low genetic diversity in the mainland are needed to test this hypothesis.

### Chemical divergences

Reproductive traits are generally assumed to be shaped by stabilizing selection to maximize encounter rates among conspecifics [36], [131]–[135], to maintain reproductive isolation barriers and to decrease the likelihood of hybridization events among syntopic sister species [31], [37]–[38]. The lower species diversity found on islands can theoretically reduce inter-specific mating opportunities (and therefore decrease the likelihood of hybridization events among sister species), which could relax the stabilising selection on courtship signals. We therefore expected an increase of MMS variability among the Corsican bumblebees due to the impoverished bumblebee fauna on the island.

Our results do not support this hypothesis since we did not observe a relaxation of the stabilizing selection on the MMS within the island. Geographic variation in courtship traits found here has been observed in several species such as moths [136]–[139], *Drosophila* flies [140], bees [141], [142]) and birds [143]. Previous studies showed that bumblebee females (ethological tests are only available for *B. terrestris*) are able to discriminate the MMS from various populations and, in the case of Corsican females, to exhibit significant preferences for the MMS from Corsican subspecies [56]. Many studies on sexual selection have largely documented that individuals often recognize and prefer to mate with individuals from “local” vs “exotic” populations (e.g. [36], [144], [145]). Preferences of bumblebee females suggest sexual selection acting on the MMS and stabilizing the Corsican MMS type. In contrast, preferences of the receiver for exotic pheromonal blend have been also observed in bees [141], flies [144] and crickets [146]. In *B. terrestris*, females prefer the MMS of exotic males inside its own subspecies, probably to minimize sibling mating [56]. We suggest that the current Corsican MMS pattern (divergence from the mainland and small inter-individuals variation) is explained by sexual selection (balance between female preferences for its own subspecies and exotic MMS) and genetic differentiation. The genetic basis of variation in chemical blends involved in chemical communication and its heritability has been demonstrated in moths (review in [147]). Genetic differentiation can thus result in altered chemical blends [39], [132], [148]–[150]. Most of these altered blends are selected against (stabilizing selection) through the sexual selection (i.e. altered blends are not recognized by receivers) [31]. Nevertheless some releasers with an altered blend can evolutionarily be tracked by receivers because of a putative wide response window of receivers (e.g. in moths [149]). Moreover, these releasers with an altered blend can spread in the population, thanks to some ecological factors (e.g. [151]), intraspecific factors strongly selected for such a change (e.g. receiver preference [132]), or stochastic events prevailed over selection [152]. In Corsican bumblebees, we expect that a recognized altered blend has spread in the population and overcome old signals through preference of the females for exotic blends before a new period of stabilizing selection [132].

### Insular species community

Like a physical landscape cluttered with visual stimuli competing for attention, the “scentscape” is muddled with odors of different types arising from a wide variety of sources [153]. Thus, in order to successfully communicate with the recipients (e.g. minimization of mismating events), the courtship signal of a species is shaped by selection to be different from others produced in the same environment [31], [38], [39]. This selection for species specific courtship signal corresponds to trap each species to a narrow range of signals different from other scents of the local scentscape (theory of adaptive landscape [152]). Changes in taxa composition of local species community or in chemical signals of species of this community can thus lead to reorganising of the local scentscape. The Corsican MMS pattern (divergence from the mainland and small inter-individuals variation) and changes in MMS interspecific distances between mainland and the island suggest that scentscape reorganization happened on the island.

### Insular inquiline species

*Bombus terrestris* and *B. vestalis-perezi*, the host-inquiline pair, are both genetically and ecologically differentiated from the mainland. These differentiations could be generated by a process of reciprocal adaptations in response to selective pressures imposed by the interacting species on each other (coevolution) [154]. Morphological and chemical adaptations to parasitism suggest a coevolution (evolutionary arms race) between *B. terrestris* and *B. vestalis* [155]–[157]. However, the similar pattern of differentiation in Corsican *B. lucorum* suggests that similar ecological constraints or stochastic events, rather than coevolution, might be responsible for observed patterns of co-differentiation [158], [159].

### Insular speciation process

The geographic differentiation of courtship traits can directly interfere in the intraspecific communication between distant populations (e.g. in moths [160] or in bumblebees [161]); the consequence can range from simple regional variation (like dialects in songbirds [162] or in moths [163] or in solitary bees [141] consisting of different relative amounts of the same key compounds) to the establishment of a reproductive (pre-zygotic) isolation barriers between populations [164].

Results on MMS of Corsican *B. lucorum* and *B. perezi* could suggest only a Corsican dialect because of insular and continental populations share the same main compounds. Further bioassays on these two taxa are needed to test this hypothesis. Among *B. terrestris*, MMS differentiation of Corsican population involves main compounds and could suggests more drastic consequences on pre-mating recognition. Behavioral bioassays support this hypothesis: *B. terrestris* females exhibit significant preferences for MMS from its own subspecies [56] even if all subspecies are able to interbreed in experimental conditions [58], [161], [165], [166]. Coppée [56] suggested an incipient allopatric speciation process for Corsican *B. terrestris* according to MMS divergence and behavioral bioassays. However, pre-mating animal communication is highly complex and often involves multiple mating cues and signal modalities [167]–[172]. Detecting speciation process driven by differentiation in reproductive traits needs a global view of the species mating recognition system [31], which is difficult to comprehend. Moreover, establishing reproductive isolation in natural conditions by divergence in reproductive traits between insulars and continentals means that a degree of subjectivity is necessary in deciding whether allopatric populations have diverged enough to prevent interbreeding [173]. Therefore, further integrative taxonomy, including on morphologic and genetic criterions [73], [174] based on an adapted species concept for the comparison of allopatric taxa (e.g. [175]), are needed in order to assess if the Corsican MMS differentiations involve pre-mating reproductive isolations.

## Conclusions

Compared to mainland populations, isolation of Corsican populations has led to genetic and MMS divergences. The Corsican taxa have presumably diverged by allopatric differentiation without genetic and demographic processes intrinsic to island populations, such as loss of genetic diversity. We hypothesize that MMS divergence in Corsican bumblebees most likely results from genetic differentiation, reinforced by insular specific sexual selection. The impoverishment of Corsican bumblebee fauna has not led to relaxing the stabilising selection on MMS. However, MMS differentiation and changes in bumblebee community in Corsica seem to have led to the reorganization of signal system on the island.

## Supporting Information

Table S1 Table of sampling. Group = Group model species; Code = Sample labels used in figures; Population = names of populations used in SMP and genetic analyses; Land: BE = Belgium, CZ = Czech Republic, DE = Germany, DK = Denmark, FR = France, GR = Greece, HU = Hungary, IT = Italy, NL = Netherlands, PL = Poland, RO = Rumania, SE = Sweden, SK = Slovakia, SL = Slovenia; Recorder : AC = Audrey Coppée, AR = Arnaud Roelandts, DM = Denis Michez, MC = Maurizio Cornalba, MT = Michael Terzo, PL = Patrick Lhomme, PR = Pierre Rasmont, SD = Simon Dellicour, SL = Sophie Lambert, TD = Thibaut De Meulemeester, TL = Thomas Lecocq; Cytb, COI, EF-1α, and PEPCK are the Genbank accession numbers for each samples.
(XLS)

Table S2 MMS data matrix (relative amounts of each compound) of *B. lucorum*.
(XLS)

Table S3 MMS data matrix (relative amounts of each compound) of *B. terrestris*.
(XLS)

Table S4 MMS data matrix (relative amounts of each compound) of *B. vestalis*.
(XLS)

Table S5 List of the identified compounds in Corsican and continental *B. lucorum*. Molecular weight (MW (m/z)), median (Med (%)), first and fourth quartiles (Q1 (%) and Q2 (%)), minimum and maximum (Min (%) and Max (%)) of the 55 identified compounds. Unknown Lucorum x are undetermined compounds.
(XLS)

Table S6 List of the identified compounds in Corsican, Sardinian and continental *B. terrestris* Molecular weight (MW (m/z)). Median (Med (%)). First and fourth quartiles (Q1 (%) and Q2 (%)). Minimum and maximum (Min (%) and Max (%)) of the 105 identified compounds. Unknown Terrestris x are undetermined compounds.
(XLS)

Table S7 List of the identified compounds in *B. perezi* and *B. vestalis*. Molecular weight (MW (m/z)). Median (Med (%)). First and fourth quartiles (Q1 (%) and Q2 (%)). Minimum and maximum (Min (%) and Max (%)) of the 56 identified compounds. Unknown Vestalis x are undetermined compounds.
(XLS)
